# Monocytes prevent apoptosis of iPSCs and promote differentiation of kidney organoids

**DOI:** 10.1186/s13287-024-03739-8

**Published:** 2024-05-03

**Authors:** Ekaterina Pecksen, Sergey Tkachuk, Cristoph Schröder, Marc Vives Enrich, Anindita Neog, Cory P. Johnson, Niko Lachmann, Hermann Haller, Yulia Kiyan

**Affiliations:** 1 Clinics for Kidney and Hypertension Disease, Hannover Medical School, Hannover, Germany; 2 Mount Desert Island Biological Laboratory, Bar Harbor, Maine USA; 3 Department of Pediatric Pneumology Allergology and Neonatology, Hannover Medical School, Hannover, Germany; 4 Fraunhofer Institute for Toxicology and Experimental Medicine ITEM, Hannover, Germany

**Keywords:** iPSCs, Kidney organoids, Apoptosis, Monocytes, iPSCs-macrophages, Autophagy

## Abstract

**Background:**

Induced pluripotent stem cells (iPSCs)-derived kidney organoids are a promising model for studying disease mechanisms and renal development. Despite several protocols having been developed, further improvements are needed to overcome existing limitations and enable a wider application of this model. One of the approaches to improve the differentiation of renal organoids in vitro is to include in the system cell types important for kidney organogenesis in vivo, such as macrophages. Another approach could be to improve cell survival. Mesodermal lineage differentiation is the common initial step of the reported protocols. The glycogen synthase kinase-3 (GSK-3) activity inhibitor, CHIR99021 (CHIR), is applied to induce mesodermal differentiation. It has been reported that CHIR simultaneously induces iPSCs apoptosis that can compromise cell differentiation. We thought to interfere with CHIR-induced apoptosis of iPSCs using rapamycin.

**Methods:**

Differentiation of kidney organoids from human iPSCs was performed. Cell survival and autophagy were analyzed using Cell counting kit 8 (CCK8) kit and Autophagy detection kit. Cells were treated with rapamycin or co-cultured with human monocytes isolated from peripheral blood or iPSCs-macrophages using a transwell co-culture system. Monocyte-derived extracellular vesicles (EVs) were isolated using polyethylene glycol precipitation. Expression of apoptotic markers cleaved Caspase 3, Poly [ADP-ribose] polymerase 1 (PARP-1) and markers of differentiation T-Box Transcription Factor 6 (TBX6), odd-skipped related 1 (OSR1), Nephrin, E-Cadherin, Paired box gene 2 (Pax2) and GATA Binding Protein 3 (Gata3) was assessed by RT-PCR and western blotting. Organoids were imaged by 3D-confocal microscopy.

**Results:**

We observed that CHIR induced apoptosis of iPSCs during the initial stage of renal organoid differentiation. Underlying mechanisms implied the accumulation of reactive oxygen species and decreased autophagy. Activation of autophagy by rapamacin and by an indirect co-culture of differentiating iPSCs with iPSCs-macrophages and human peripheral blood monocytes prevented apoptosis induced by CHIR. Furthermore, monocytes (but not rapamycin) strongly promoted expression of renal differentiation markers and organoids development via released extracellular vesicles.

**Conclusion:**

Our data suggest that co-culturing of iPSCs with human monocytes strongly improves differentiation of kidney organoids. An underlying mechanism of monocytic action implies, but not limited to, an increased autophagy in CHIR-treated iPSCs. Our findings enhance the utility of kidney organoid models.

**Supplementary Information:**

The online version contains supplementary material available at 10.1186/s13287-024-03739-8.

## Introduction

Several protocols for differentiating human iPSCs to 3D structures termed ‘organoids’ resembling the anatomical structure of the corresponding organ have been developed [1]. As understanding of kidney embryonic development progressed, several approaches towards the differentiation of stem cells to kidney organoids have been reported [2–5]. Generally, these approaches rely on the same signaling pathways and achieve similar development of kidney organoids. Metanephric kidney develops from reciprocally interacting metanephric mesenchyme and the nephric duct [6]. Both cell populations derive from intermediate mesoderm that takes its origin from late-stage primitive streak. Nephric duct is derived from anterior and metanephric mesenchyme from posterior intermediate mesoderm [2, 7]. Nephron progenitors of metanephric mesenchyme produce factors including Glial Cell Line-derived Neurotrophic Factor (GDNF), Fibroblast growth factor (FGF), and Bone morphogenetic protein (BMP) ligands to promote branching morphogenesis of ureteric bud – outgrowth of the nephric duct invading the metanephric mesenchyme. In turn, BMP, FGF and canonical Wingless/Integrated (Wnt) signals produced by ureteric bud support proliferation and maintenance of nephron progenitors [8]. Several transcriptome studies have been performed to address robustness and reproducibility of kidney organoids differentiation protocols and revealed batch-to-batch variability and differences in the degree of differentiation between the compared protocols. Furthermore, the main problem of kidney organoids in vitro remains their immaturity. Transcriptional similarities with human kidney of trimester I have been reported [3]. In addition, recent single-cell transcriptome studies suggested that there are significant differences between cell types in organoids and human fetal tissues [9–11]. Also, morphological structure and functional maturation of kidney organoids need to be improved [12]. Despite these limitations, kidney organoids have found an application for studying kidney development, modeling renal diseases, and for drug screening. Therefore, improvements in the differentiation protocols should enable wider applications of renal organoids. One of the approaches to improve kidney organoids differentiation in vitro is to include in the in vitro system other cell types that are important for kidney organogenesis in vivo, primarily macrophages [13, 14] and vascular cells [15, 16].

Macrophages are inborn phagocytes and are important to the early stages of organ development. Macrophages can be detected in a renal interstitium even before renal organogenesis [13]. However, the role of macrophages is not limited to the clearance of apoptotic cells and fighting the infection. Tissue-resident macrophages maintain homeostasis, conduct disease responses, and organize tissue and organ repair [15, 17]. During renal development, macrophages regulate fate and restrict the nephron progenitors population, participating in signaling, conducting trophic function, and promoting vasculature interconnections [13, 14]. Recent work showed that kidney tissue macrophages originate from fetal liver monocytes [18]. Depletion of renal tissue macrophage pool in the adult kidney leads to recruitment of circulating monocytes that acquire specific phenotypes under the influence of renal niche [18].

The influence of monocytes on the differentiation of iPSCs-kidney or other organ organoids in vitro has not been addressed yet. We reasoned that macrophages could play a role in the differentiation of iPSCs along the renal development not only in vivo but also in our in vitro setting. To investigate the crosstalk between differentiating renal progenitors and monocytes, we established a co-culture system of human monocytes or iPSCs-derived macrophages and kidney organoids. We found that monocytes promote iPSCs survival during initial CHIR-induced differentiation via the release of extracellular vesicles (EVs) and induction of autophagy. Furthermore, the superior survival resulted in more effective kidney organoids differentiation.

Autophagy is a pathway of lysosomal degradation of damaged internal cellular components such as proteins and organelles [19]. It is a fundamental process for the maintenance of postmitotic tissue homeostasis as well as for controlling stem cells’ fate [20]. Autophagy can be activated in response to various stresses including oxidative stress, DNA damage, endoplasmic reticulum stress, and starvation to promote cell survival [20]. In addition, autophagy can be activated by extracellular signals. Thus, tumor-associated macrophages induced autophagy in cancer cells by an unknown mechanism [21].

Mesodermal lineage differentiation induction by the glycogen synthase kinase-3 (GSK-3) activity inhibitor, CHIR99021 (CHIR), is the common initial step of all reported protocols for iPSCs differentiation to kidney organoids. Reports showed that CHIR induces dose-dependent apoptosis in mouse embryonic cells [22] and that massive apoptosis takes place during initial stages of human iPSCs differentiation towards cardiogenic mesoderm lineage [23, 24]. Cell death is an important aspect of differentiation and development [25]. During iPSCs differentiation in vitro, blockade of apoptosis prevented mesodermal commitment [23]. However, excessive apoptosis hurdles efficient iPSCs differentiation. One of the approaches to regulate cell death and to improve cardiomyocyte differentiation was the activation of autophagy by application of the Mammalian target of rapamycin (mTOR) inhibitor, rapamycin [26]. We also applied rapamycin and observed better iPSCs survival during CHIR stimulation. However, no improvement of organoid differentiation was induced by this approach.

Our data show that by adding macrophages we could induce a positive effect on renal organoid development. Our novel approach promotes the successful application of renal organoids for disease modeling and drug development.

## Material and methods

### Materials

All reagents, kits, antibodies, RT-PCR primers used in the study are listed in the Additional file 2 Table S1.

### Cells and cell culture

Human Episomal iPSCs Line (ThermoFisher Gibco, A18945) was used in the study. Induced pluripotent stem cells (iPSCs) were cultured using StemFlex medium (Gibco) on Geltrex (Gibco)-coated cell culture plates. ED-iPSCs cell line was a kind gift of Dr. Aloise Mabondzo (CEA, Institute Joliott, Paris, France). Cell lines authentication was performed at Eurofins (https://eurofinsgenomics.eu/de/). Results are shown in Additional file 3 Table S2. No matching cell line was found using Short Tandem Repeats search of the DSMZ database (https://celldive.dsmz.de/str/search) [27].

Human peripheral blood CD14 + CD16– monocytes were isolated from buffy coats obtained from the German Red Cross (DRK-Blutspendedienst NSTOB, Springe, Germany) using a Classical Monocyte Isolation Kit (Miltenyi Biotec). The methods were in accordance with relevant guidelines and regulations. The functionality of monocytes has been tested by their ability to differentiate into macrophages in the presence of Macrophage colony-stimulating factor (M-CSF) and then polarize into M1- and M2-like macrophages [28]⁠. Monocytes were treated with 20 ng/mL M-CSF (Peprotech) for 7 days and then polarized during 24 h. For M1-like polarization, cell stimulation with 20 ng/mL Interferon gamma (IFNγ) (Peprotech) and 20 ng/mL Lipopolysaccharide (LPS) (Sigma) was used, for M2-like polarization 20 ng/mL of Interleukin 4 (IL-4) (Peprotech) was used. Expression of Interleukin 6 (IL-6), Interleukin 10 (IL-10), and Tumor Necrosis Factor alpha (TNFɑ) was used to characterize polarization of macrophages. Only co-cultures with functionally differentiated monocytes have been used for further analysis.

Human iPSCs-derived macrophages were differentiated as previously described [29, 30].

### iPSCs-kidney organoid differentiation

We performed kidney organoid differentiation according to the protocol reported by Morizane et al. [4] with modifications. We applied an adherent culture differentiation protocol. Cells were differentiated in Basal Medium (BM) consisting of Advanced RPMI 1640 Medium (Gibco) with 200 µM L-Glutamine and 0.5% KnockOut Serum Replacement (Gibco). iPSCs were seeded at a density of 0.75 × 10^6^ cells/cm^2^. On the following day, 10 µM CHIR-99021 (Selleckchem) and 5 ng/ml noggin (PeproTech) were applied for 4 days with refreshment of the medium after 2 days. On Day 4 medium was changed to BM supplemented with 10 ng/ml Activin A (PeproTech) where cells were cultured for 3 days. Then 10 ng/ml Fibroblast growth factor 9 (FGF9) Protein (R&D Systems,) was added in BM for 2 days. On Day 9 of the differentiation supplemented with 10 ng/ml FGF9 and 3 µM of CHIR BM medium was refreshed. From day 11 to 14 was used BM with 10 ng/ml FGF9. From day 14 until day 28 day of differentiation, organoid progenitors were switched to BM, which was refreshed every 2 days. A discrete aggregate of progenitor cell, recognizable in bright-field microscopic image as a cluster of 3D nephron-like structures was counted as a single organoid.

On Day 0 of kidney organoids differentiation iPSCs-derived macrophages or human monocytes were placed at a density of 1 × 10^5^ or 2 × 10^5^ as indirect co-culture in Thincert cell culture inserts with pores diameter 0.4 µm for 24 or 6-well plate, respectively. The volume of medium has been adjusted proportionally to the total cell number. Rapamycin was added at a concentration of 0.4 µM.

### Kidney organoid fixation and staining

For fluorescence microscopy, kidney organoids were fixed for 1 h in 4% paraformaldehyde in Dulbecco's Phosphate-Buffered Saline (DPBS) for 60 min at room temperature. The fixation solution was removed, organoids were washed 3 × with 0.1% Triton X-100, blocked in DPBS containing 5% goat serum, 5% donkey serum with 1% Triton X-100 in 5% BSA for 1 h twice, and incubated overnight with primary antibodies at 4°C. Then they were 3 × washed with 1%Triton X-100 in DPBS for 1 h and incubated with secondary antibodies overnight at 4°C or at room temperature for 3 h. To clear the organoids was used 20% Formamide/DPBS vol/vol for 30 min, 40% Formamide/DPBS vol/vol for 30 min, 80% Formamide/DPBS vol/vol for 1 h, 95% Formamide/DPBS vol/vol for 2 h. Images were taken using oil-immersed x 20 objective at the Research Core Unit for Laser Microscopy at Hannover Medical School. ImageJ software was used for creating plot profile, sum of z-stacks and 3D viewer plugin was used for creating animations.

### Cell assays

Live cell numbers were quantified using Cell counting kit 8 (CCK8, Dojindo). Briefly, inserts with monocytes were removed from the wells and 10 µl of reagent per 100 µl of medium was added to the wells. Cells were placed in the incubator. After 1 h absorbance was measured at 450 nm. Then, cells were rinsed with pre-warmed PBS and medium was replaced.

The ROS levels were assessed using 5- (and 6-)Carboxy-2',7'-Dichlorodihydrofluorescein-Diacetate (Carboxy-H_2_DCFDA, ThermoFisher) dye. CellROX green reagent (ThermoFisher) was used as recommended by the supplier, and MitoSOX green reagent (ThermoFisher) was used as recommended by the supplier to detect mitochondrial superoxide production. For normalization of the cell number per well, cells were fixed with 4% paraformaldehyde for 10 min at room temperature, then 50 µl of 0.5% crystal violet/H_2_O solution was added to each well, cells were stained for 30 min on the orbital shaker, then washed 3 times with 200 µl PBS/well. After the last wash, 200 µl of 1% SDS solution was added, and absorbance was read at 570 nm.

Biotracker 488 Green Mitochondria Dye (Millipore) was used to quantify mitochondria content in the cells using Nunc 96-Well Optical Coverglass Bottom plates and TECAN Multiplate Reader (Tecan Group). Normalization of cell number /well was performed as described above. We used Cyto-ID Autophagy Detection Kit (ENZO Life Sciences) as recommended by the supplier to detect the process of autophagy.

EVs from monocyte conditioned medium were concentrated using the protocol described recently [31] with slight modifications. Briefly, EVs were isolated from conditioned media from six transwell inserts each containing 2,5 × 10^5^ monocytes. Cells were incubated either in BM, or BM containing 10 µM CHIR and 5 ng/mL noggin, or from monocytes and iPSCs co-cultured in BM containing 10 µM CHIR and 5 ng/mL noggin for 3 and 6 days. Conditioned medium was centrifuged at 500 g to remove cell debris. Then, conditioned medium was mixed in a 1:1 ratio with a concentrating solution containing 24% of polyethylenglycol (PEG) with an average molecular weight of 6000 and 1 M sodium chloride. After overnight incubation, EVs were enriched by centrifugation at 10 000 g for 1 h at 4 °C. Supernatant was then removed and EVs were dissolved in BM and applied to three wells of iPSCs at Day 0 of renal organoid differentiation protocol. 10 µM CHIR and 5 ng/mL noggin were added to the iPSCs simultaneously with EVs. The experiment has been independently repeated two times.

L-lactate was analyzed using an assay Kit from Promocell.

### Cell lysis and western blotting

For western blotting cells were washed with ice-cold PBS, placed on ice and lysed using RIPA lysis buffer containing 25 mM Tris–HCl, pH 7.6, 150 mM NaCl, 1% NP-40, 1% sodium deoxycholate and 0.1% SDS with added inhibitors of proteases and phosphatases (1 mM phenylmethylsulfonyl fluorid, 10 µg/ml aprotinin, 10 µg/ml Leupeptin, 0.3 mM sodium orthovanadate). Cells were scraped and allowed to lyse on ice for 10 min. SDS-PAGE electrophoresis and semi-wet protein transfer on PVDF membrane were performed. Antibodies used in the study are listed in the Additional file 2 Table S1. Western blotting membranes were imaged and quantified using VersaDoc and QuantityOne software (Bio-Rad Laboratories, Inc.). Uncropped western blotting images are shown in Additional file 1 Figs. S2 and S3.

### RT-PCR

We isolated RNA from renal organoids using Qiagen RNeasy mini kit. Approximately 100 organoids were used for each RNA probe. The list of TaqMan gene expression assays from ThermoFisher Scientific used in the study is given in the Additional file 2 Table S1. LightCycler480 RNA Master Hydrolysis Probes (Roche Diagnostics GmbH) and Roche LightCycler96 were used.

### Statistics

All data were obtained with at least 3 biological replications. Data are presented as mean ± standard deviation (SD). Multiple comparisons were analyzed by ANOVA with Tukey post hoc test. *P* values < 0.05 were considered statistically significant. GraphPad Prism 8.3.0 (GraphPad Software) was used for data analysis. *means *P* values less than 0.05; **means *P* values less than 0.01; ***means *P* values less than 0.001.

## Results

### CHIR induced apoptosis of iPSCs

**Fig. 1:**
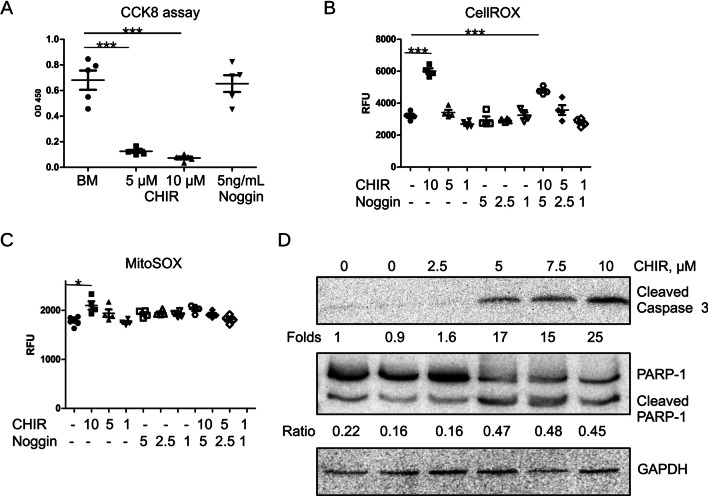
CHIR induces apoptosis of iPSC. **A** iPSC live cell number after 48 h of stimulation with different concentrations of CHIR or noggin was assessed using CCK8 kit. **B** Cellular level of ROS in the presence of different concentrations of CHIR and noggin separately and together was analyzed 24 h after their addition using CellRox green reagent. Concentrations of CHIR is in µM, concentrations of Noggin is in ng/mL. **C** Mitochondrial superoxide production in the presence of different concentrations of CHIR and noggin separately and together was analyzed 24 h after their addition using MitoSOX green reagent. Concentrations of CHIR is in µM, concentrations of Noggin is in ng/mL. **D** iPSC treated with different concentrations of CHIR in the presence of 5 ng/ml Noggin were lysed and expression of cleaved caspase 3, cleaved PARP-1 was assessed by western blotting. Full-length blots are shown in Additional file 1 Fig. S2

Several groups reported that CHIR induced apoptosis during iPSCs differentiation [24, 26]. Since we have also observed strong cell death during iPSCs stimulation with CHIR and noggin, we first quantified cell survival using a CCK8 kit. We applied 10 µM CHIR and 5 ng/mL noggin as these were optimal concentrations for the differentiation of renal organoids [4]. Cells were stimulated with CHIR and noggin for 48 h. CHIR at a concentration of 10 µM and even at a lower concentration of 5 µM but not noggin at 5 ng/mL induced a strong decrease of live cell number (Fig. 1A). Next, we assessed whether or not cell stimulation with CHIR results in the accumulation of reactive oxygen species (ROS). We treated cells with varying concentrations of CHIR and noggin alone and in combination and then applied CellROX green fluorogenic indicator to detect the level of cellular ROS. The dye becomes fluorescent and binds to DNA after being oxidized by O2^−^ and/or •OH in the living cells. Data presented in Fig. 1B show that CHIR induced oxidative stress in the cells in a concentration-dependent manner, whereas the application of noggin did not cause any ROS production. Accordingly, cell treatment with CHIR and noggin simultaneously induced ROS similarly as CHIR alone. To clarify the source of cellular ROS, we used the same experimental setting as above and applied mitochondrial superoxide indicator MitoSOX. Our data showed that mitochondria also produced superoxide in response to stimulation with CHIR (Fig. 1C). Though CHIR-induced superoxide increase was statistically significant, the degree of activation was significantly less than the one detected by CellROX reagent.

Increased oxidative stress resulted in turn in cell apoptosis. Expression of cleaved caspase 3 and cleaved Poly [ADP-ribose] polymerase 1 (PARP-1) was concentration-dependently increased by CHIR (Fig. 1D). However, decreasing CHIR concentration interfered with iPSCs differentiation and strongly inhibited the formation of kidney organoids (data not shown).

### Rapamycin and human monocytes promote survival of iPSCs via activation of autophagy

We selected two approaches to look for possible interventions that could decrease cell death during CHIR stimulation. We based our first hypothesis on the report that upon activation of WNT signaling, β-catenin negatively regulates autophagy [32]. Since inhibition of autophagy was reported to induce cell death in iPSCs [33] and activation of autophagy decreased apoptosis and promoted cardiomyocyte differentiation [26] we decided to apply the mTOR inhibitor, rapamycin. Secondly, since macrophages co-cultured with hepatocellular carcinoma cells could induce autophagy in tumor cells and promote their survival [21]⁠, we performed differentiation of iPSCs using indirect transwell co-culture with human monocytes and iPSCs-macrophages. Classical human CD14 + /CD16- monocytes were isolated from buffy coats using negative selection magnetic sorting. The functionality of monocytes has been routinely tested by their ability to differentiate into macrophages in the presence of M-CSF and then polarize into M1- and M2-like macrophages. For M1-like polarization, IFNγ and LPS stimulation was used, for M2-like polarization IL-4 was used. Expression of IL-6, IL-10, and TNFɑ was used to characterize the polarization of macrophages (Additional file 1 Fig. S1A). Despite some variability between cells isolated from different donors, differentiated macrophages demonstrated similar pattern of expression. To determine the optimal ratio of monocytes and iPSCs, we performed co-culture using different numbers of monocytes (Additional file 1 Fig. S1B). We selected the ratio of 2.5 × 10^5^ monocytes / 10^7 ^iPSCs because increasing the numbers of monocytes did not result in further improvement of their effect on iPSCs survival.

**Fig. 2:**
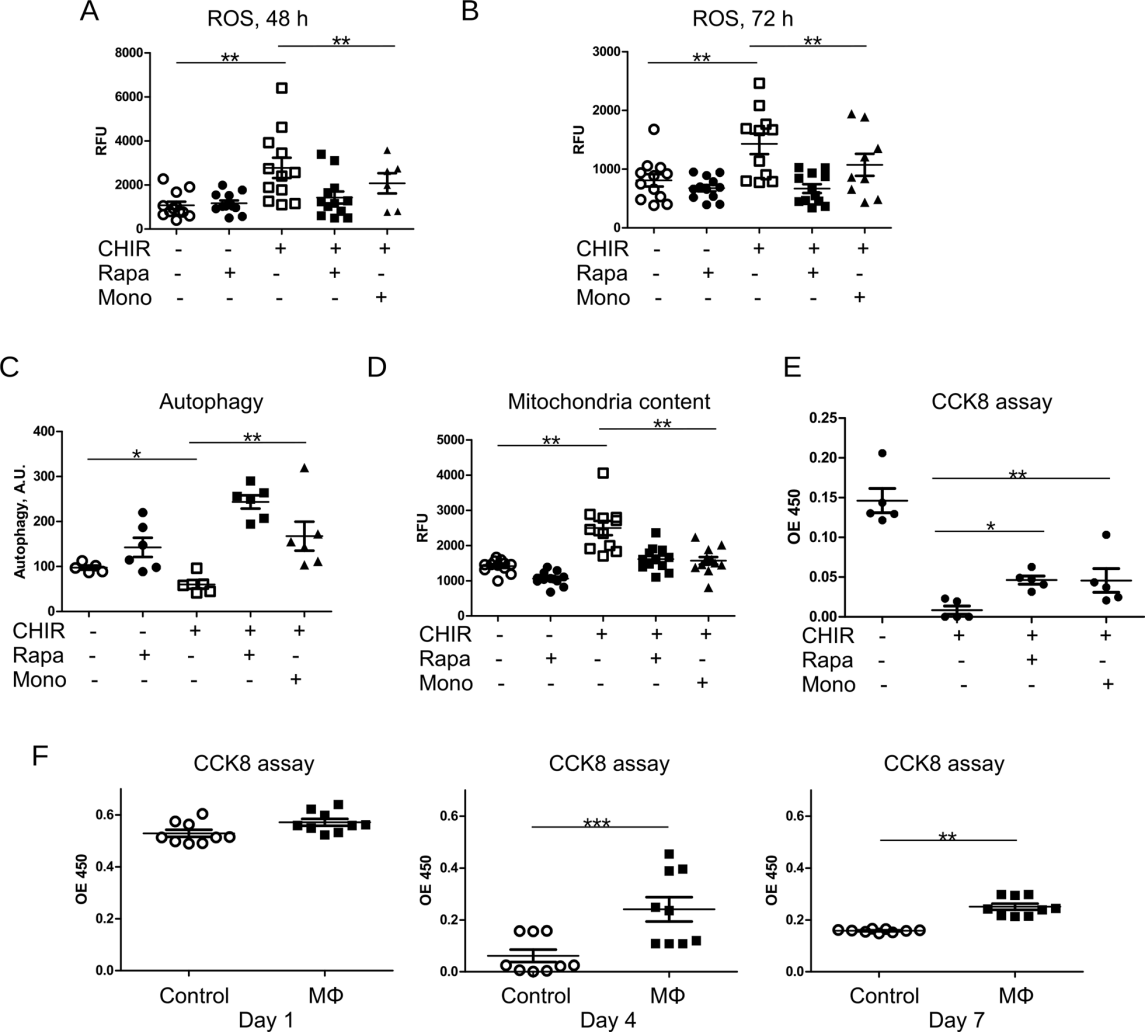
Rapamycin and monocytes rescue iPSC from apoptosis via activation of mitophagy. **A**, **B** ROS accumulation in the presence of 10 µM CHIR was measured using Carboxy-H2DCFDA dye after 48 (**A**) and 72 (**B**) hours of cell stimulation. 5 ng/mL Noggin was added to all cells. Rapamycin (Rapa) and monocytes (Mono) were applied as indicated. After measurement of fluorescence, cells were fixed and stained using crystal violet for normalization of cell number. **C** Autophagy in iPSC cells treated as indicated was assessed using Autophagy detection kit after 48 h. 5 ng/mL Noggin was added to all cells. Normalization of cell number was performed as in **A**. **D** Mitochondria content was assessed using Mitotracker green after 48 h. Normalization of cell number was performed as in **A**. **E** Survival of iPSC in the presence of Rapa and monocytes has been determined using CCK8 kit. **F** CCK8 assay was performed on ED-iPSC cell line stimulated with 10 µM CHIR and 5 ng/mL Noggin in mono-culture (Control) and in co-culture with iPSC-derived macrophages (MΦ) on Day 1, 4 and 7

Next, we analyzed the levels of ROS in cells treated with rapamycin and in co-culture with monocytes. Since we showed before that superoxide production by mitochondria was only slightly increased by cell treatment with CHIR, we applied H_2_DCFDA ROS indicator for these experiments. H_2_DCFDA has wide specificity and detects hydroxy, peroxy, and other ROS in the cell. We observed that both, rapamycin and monocytes, significantly decreased production of ROS (Fig. 2A, B).

Autophagy was analyzed using an Autophagy detection kit that selectively labels accumulated autophagic vacuoles. We showed that both, rapamycin application and co-culture with monocytes promoted autophagy (Fig. 2C). It was reported recently that despite increased mitochondrial activity, mitochondrial content is decreased in mesoderm in comparison to undifferentiated iPSCs [34]⁠. Since mitophagy, the degradation of mitochondria by the process of autophagy, represents the main mechanism of mitochondria quality control [35]⁠, we assessed the accumulation of mitochondria using mitotracker green (Fig. 2D). The CHIR-induced increase of mitochondria content was decreased by the addition of rapamycin or co-culturing with monocytes. Accordingly, cell survival was improved (Fig. 2E).

**Fig. 3:**
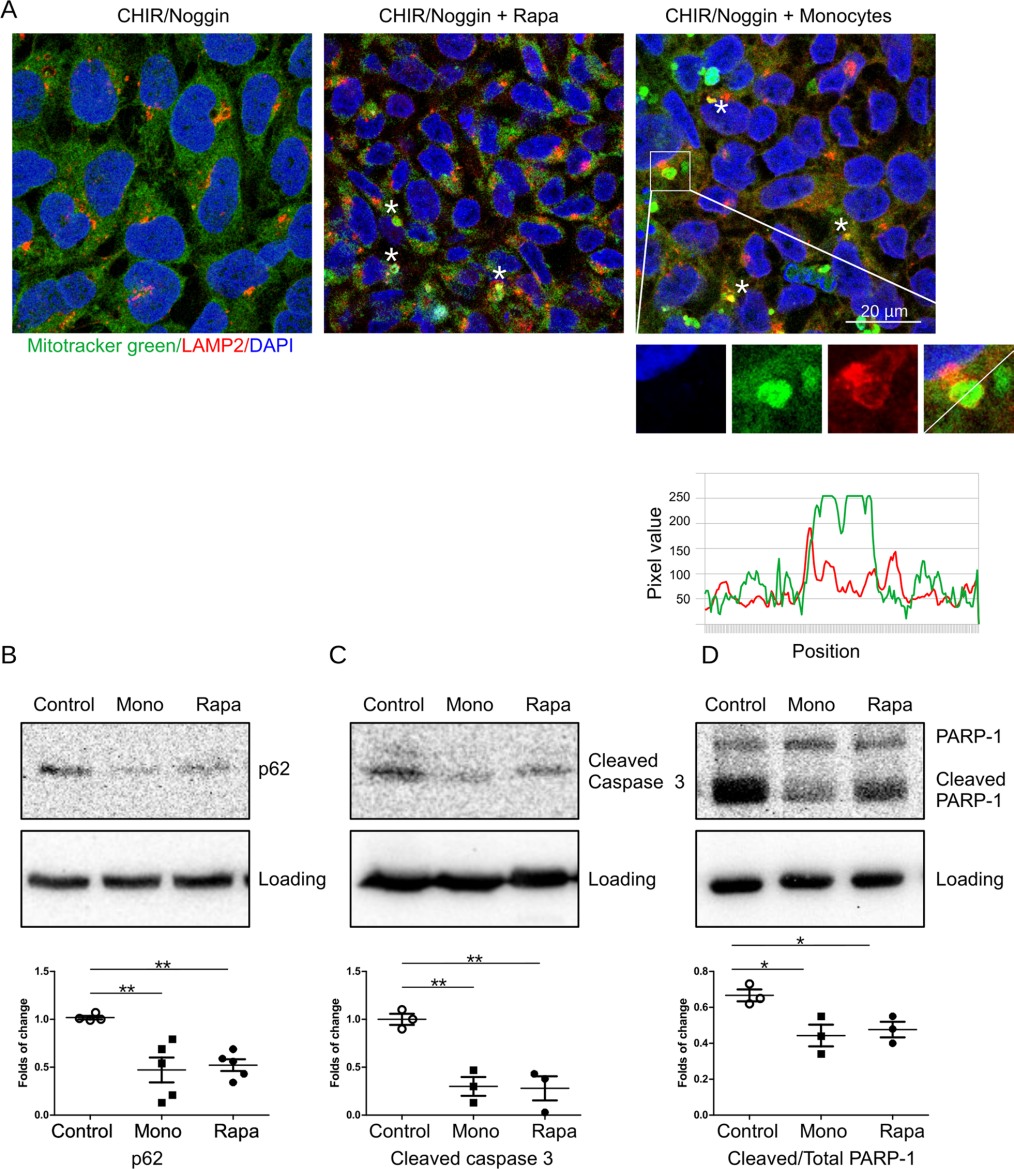
Rapamycin and monocytes rescue iPSC from apoptosis **A** Cells treated with 10 µM CHIR and 5 ng/mL Noggin for 48 h were fixed and stained using Mitotracker green and LAMP-2 antibody. Co-localization of Mitotracker green and LAMP-2 is shown by white asterisk. Higher magnification images of an autophagosome and Plot Profile of green and red fluorescence created using ImageJ software are shown in bottom panels. **B** Expression of autophagy marker p62, cleaved caspase 3 (**C**) and cleaved PARP-1 (**D**) in iPSC cells treated for 48 h with CHIR and Noggin was assessed by western blotting. The upper panels show typical western blotting picture. The lower panels show quantification for at least three independent experiments. Full-length blots are presented in Additional file 1 Fig. S2

Furthermore, we performed a CCK8 assay using different iPSCs cell lines and co-cultured the cells with human iPSCs-derived macrophages during the differentiation to kidney organoids. Our data showed (Fig. 2F) that co-culturing with iPSCs-macrophages also strongly improved cell survival. We also observed that rapamycin and monocytes promoted a formation of autophagosomes containing mitochondria and their fusion with lysosomes. We stained cells with mitotracker green and Lysosome-associated membrane protein 2 (LAMP2) as a lysosomal marker and performed confocal microscopy (Fig. 3A). We detected co-localization of mitochondria and lysosomes in cells treated with rapamycin and in co-culture with monocytes. Fluorescence plot profiles created using ImageJ software showed localization of LAMP2 around mitochondria suggesting the fusion of these organelles (Fig. 3A). Expression of apoptosis markers cleaved caspase 3 and cleaved PARP-1 in CHIR-treated iPSCs was assessed by western blotting (Fig. 3B–D). Apoptosis was decreased by both, rapamycin and in co-culture with monocytes. Decreased content of Sequestosome-1/ Ubiquitin-binding protein p62 (p62) in rapamycin-treated iPSCs and cells co-cultured with monocytes confirmed activation of autophagy.

Together these data imply that autophagy was decreased during iPSCs stimulation with CHIR which led to the accumulation of cell damage and death. We conclude that stimulating autophagy by rapamycin or via co-culturing of differentiating iPSCs with monocytes, improved cell survival.

### Mechanism of monocyte action

**Fig. 4:**
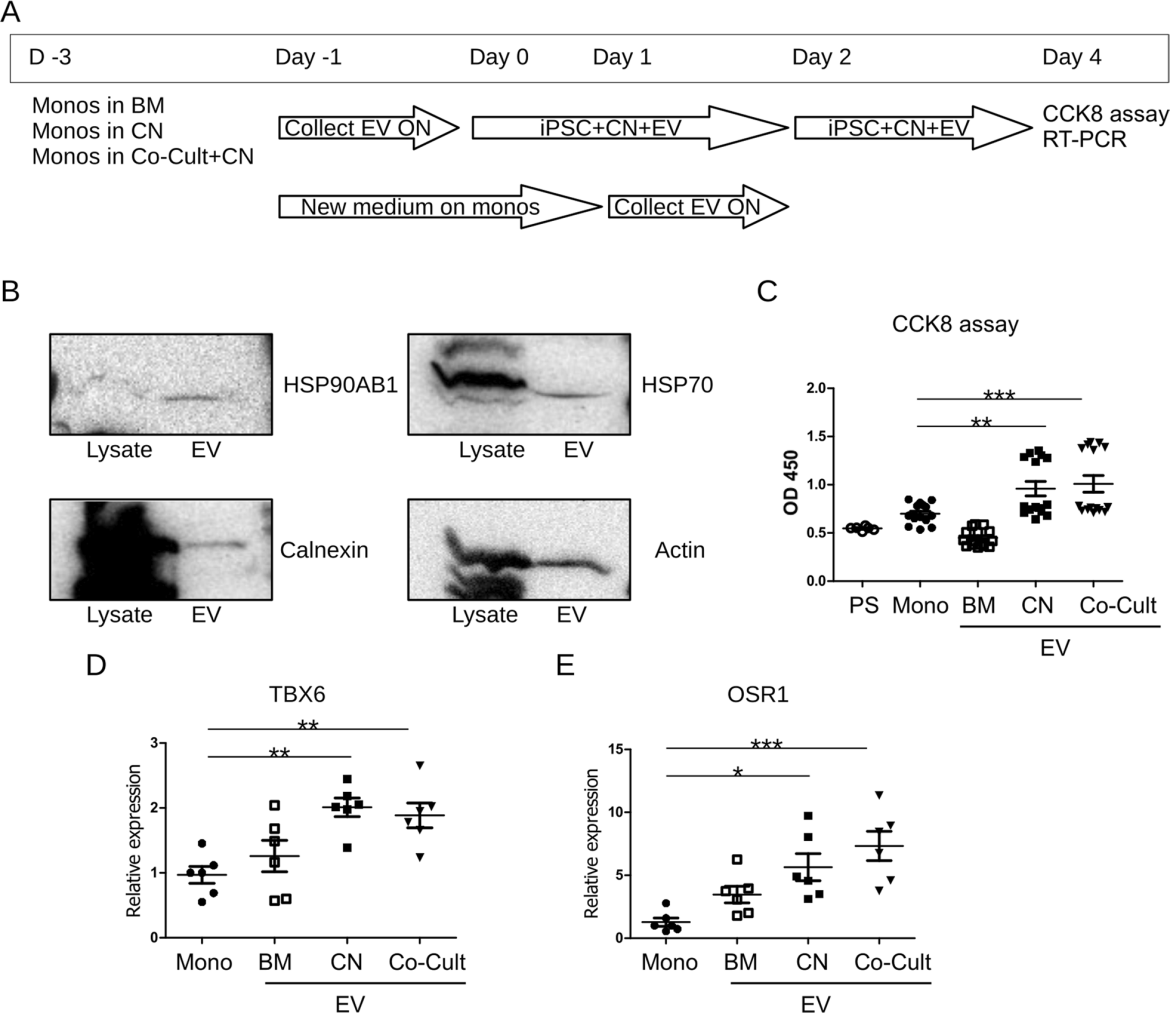
Monocytes-released EVs promote survival and differentiation of iPSC. **A** Schematic presentation of experimental design. On Day -3, monocytes (Monos) were seeded in the inserts in (i) BM; (ii) BM containing 10 µM CHIR and 5 ng/mL noggin (CN), and (iii) in coculture with iPSC in BM supplemented with CHIR and noggin (Co-cult). After 48 h (Day -1), medium from the well was collected, cell debris were removed by centrifugation, and EVs were precipitated overnight (ON). Monocytes were given the same medium (i-iii) once again. On Day 0, exosomes were added to iPSC in BM containing 10 µM CHIR and 5 ng/mL noggin. On Day, 1 EVs were harvested from monocytes and precipitated overnight (ON). On Day 2, exosomes were added to iPSC during medium refreshing with BM containing 10 µM CHIR and 5 ng/mL noggin. On Day 4, CCK8 assay was performed and RNA was extracted from cells for RT-PCR. **B** Western blotting was performed to confirm the presence of EVs marker proteins in the isolated EVs fraction. Uncropped gels are shown in Additional file 1 Fig. S3. **C** CCK8 assay was performed on iPSC differentiated in the presence of monocytes-derived EVs on Day 4. (PS)—precipitation solution control. (Mono)—mono-cultured iPSC. **D**, **E** Relative expression of late primitive streak marker TBX6 (**D**) and posterior intermediate mesoderm marker OSR1 (**E**) by iPSC differentiated in the presence of monocytes-derive EVs was assessed by RT-PCR on Day 4

EVs are membrane vesicles released by cells to deliver proteins, metabolites, nucleic acid and other bioactive molecules to recipient cells [36]. EVs are recognized as one of the key mechanisms of intercellular communications and important regulators of multiple biological processes including development. Classical EVs include exosomes, microvesicles, and apoptotic bodies, though recent works suggest the existence of further EVs types [36]⁠. Several methods of EVs isolation have been developed. Recently, PEG-based precipitation method has proven to be efficient in EVs isolation from various sources including cell culture conditioned medium [31, 37]⁠. Monocytes have also been reported to release bioactive EVs that can affect other cell types [38]⁠. To test whether monocytes release EVs to improve survival and differentiation of iPSCs, we incubated monocytes in BM, BM supplemented with 10 µM CHIR and 5 ng/mL noggin, and in co-culture with iPSCs in the presence of CHIR and noggin for 2 days. Experimental setting is schematically shown in Fig. 4A. Then, EVs were isolated from conditioned medium as described in Methods. Isolated EVs fraction was tested on the presence of exosomal marker Heat shock protein 90 alpha family class B member 1 (HSP90AB1) and Heat shock protein 70 (HSP70), endoplasmic reticulum marker Calnexin, and actin (Fig. 4B). After confirming that isolated fractions contain EVs from monocytes, we added EVs to iPSCs on Day 0 of the renal organoid differentiation protocol simultaneously with 10 µM CHIR and 5 ng/mL noggin. On Day 2 medium change on differentiating organoids has been performed and the second portion of monocytes-secreted EVs was added to the cells. CCK8 assay was performed on Day 4 of differentiation (Fig. 4C). To make sure that traces of the EVs precipitation reagent, can affect cell survival, we performed a control and showed that it does not affect on cells (Fig. 4C). EVs from monocytes incubated in BM slightly decreased survival of iPSCs though this difference has not reached statistical significance. On the contrary, EVs from CHIR- and noggin-treated monocytes and from co-cultured monocytes strongly improved the survival of differentiating iPSCs and the values of rescue effect were very similar. We have also performed RT-PCR for the expression of the Late Primitive Streak Marker T-Box Transcription Factor 6 (TBX6) [4] since cells at Day 4 of differentiation should demonstrate the expression of this gene (Fig. 4D). We observed that not only survival but also differentiation of iPSCs was improved by EVs isolated from CHIR- and noggin-treated as well as co-cultured monocytes. Furthermore, we have also demonstrated that the expression of posterior intermediate mesoderm marker odd-skipped related 1 (OSR1) was increased by the monocytes-derived EVs already at day 4 (Fig. 4E). Since we observed that EVs from co-cultured monocytes demonstrate similar effects on iPSCs survival and expression of differentiation markers as EVs form monocytes treated with CHIR and noggin in mono-culture, we concluded that monocytes released EVs in response to CHIR and noggin and this effect is not further improved by the presence of iPSCs at least during the first days of differentiation.

Taking together, our data show that in response to CHIR and noggin treatment monocytes release EVs that in turn exert anti-apoptotic and pro-survival effects on iPSCs and promote their differentiation to renal organoids.

During the differentiation of iPSCs metabolic reprogramming from aerobic glycolysis to oxidative phosphorylation is taking place. Since the content of L-lactate in conditioned medium reflects aerobic glycolysis, we measured L-lactate using a colorimetric assay kit. As shown in Additional file 1 Fig. S1C, we have not observed any differences in the concentration of L-lactate in cells treated with exosomes from monocytes, at least during the initial stages of differentiation.

### Monocytes strongly improve iPSCs differentiation to renal organoids.

**Fig. 5:**
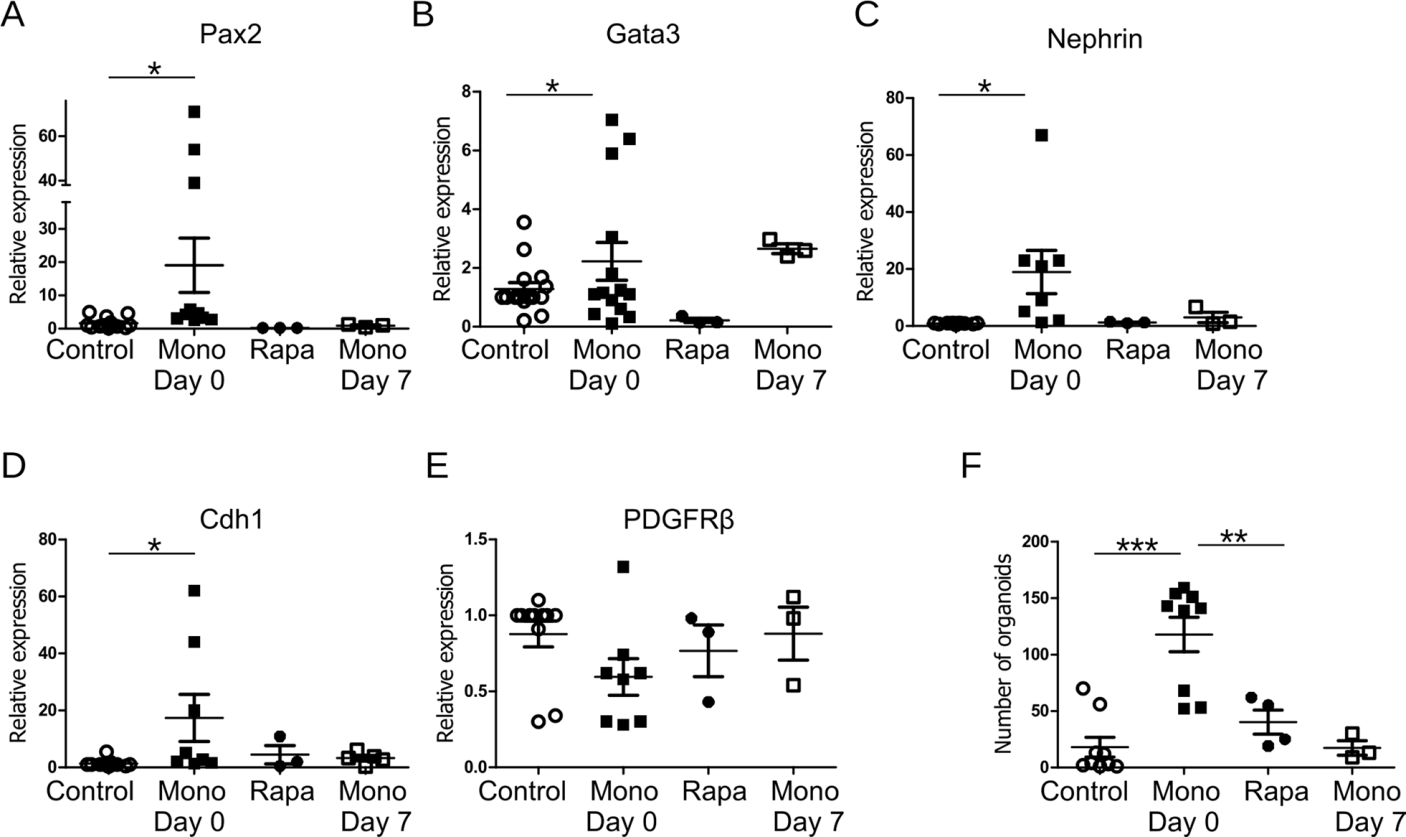
Monocytes promote differentiation of renal organoids. iPSC differentiation to renal organoids has been performed in the transwell co-culture with human monocytes from Day 0 (Mono D 0) or Day 7 (mono Day 7) of the differentiation protocol and in the presence of rapamycin (Rapa). Expression of renal markers (**A**–**E**) was assessed by TaqMan RT-PCR on Day 28 of the protocol. **F** Number of organoids per area unit was quantified on Day 28 of the protocol

To further characterize the effects of rapamycin and monocytes on the differentiation of iPSCs towards renal organoids, we applied rapamycin during the first 4 days of CHIR-induced cell differentiation. Co-culture of iPSCs with monocytes was performed during the whole protocol. In order to understand, at what stage of organoid development the effects of monocytes are important, co-culture with monocytes was also performed starting from Day 7 of the differentiation protocol. Expression of E-Cadherin, Nephrin, Paired box gene 2 (Pax2), and GATA Binding Protein 3 (GATA3) was assessed by TaqMan RT-PCR after completion of protocol from several independent experiments. We observed a robust increase of renal marker expression in cells co-cultured with monocytes during the whole protocol (Fig. 5A–E). On the contrary, co-culturing from Day 7 did not significantly improve organoids differentiation (Fig. 5A–E). Expression of some stroma-related markers like platelet derived growth factor receptor beta (PDGFRβ) was not affected by co-culturing with monocytes. Though rapamycin application increased survival during the first four days, treatment with rapamycin during that period failed to improve the differentiation of kidney organoids.

We quantified the number of organoids after 4 weeks of differentiation in mono- and co-culture with monocytes and with and without the presence of rapamycin. Monocytes but not rapamycin strongly promote renal differentiation of iPSCs (Fig. 5F). These data suggest that, even though improvement of autophagy at the first step of renal organoid differentiation can improve cell survival, effects of monocytes during the differentiation are not limited to the activation of autophagy. Furthermore, if co-culturing has begun from Day 7 of the protocol, monocytes failed to improve differentiation of iPSCs to kidney organoids.

**Fig. 6:**
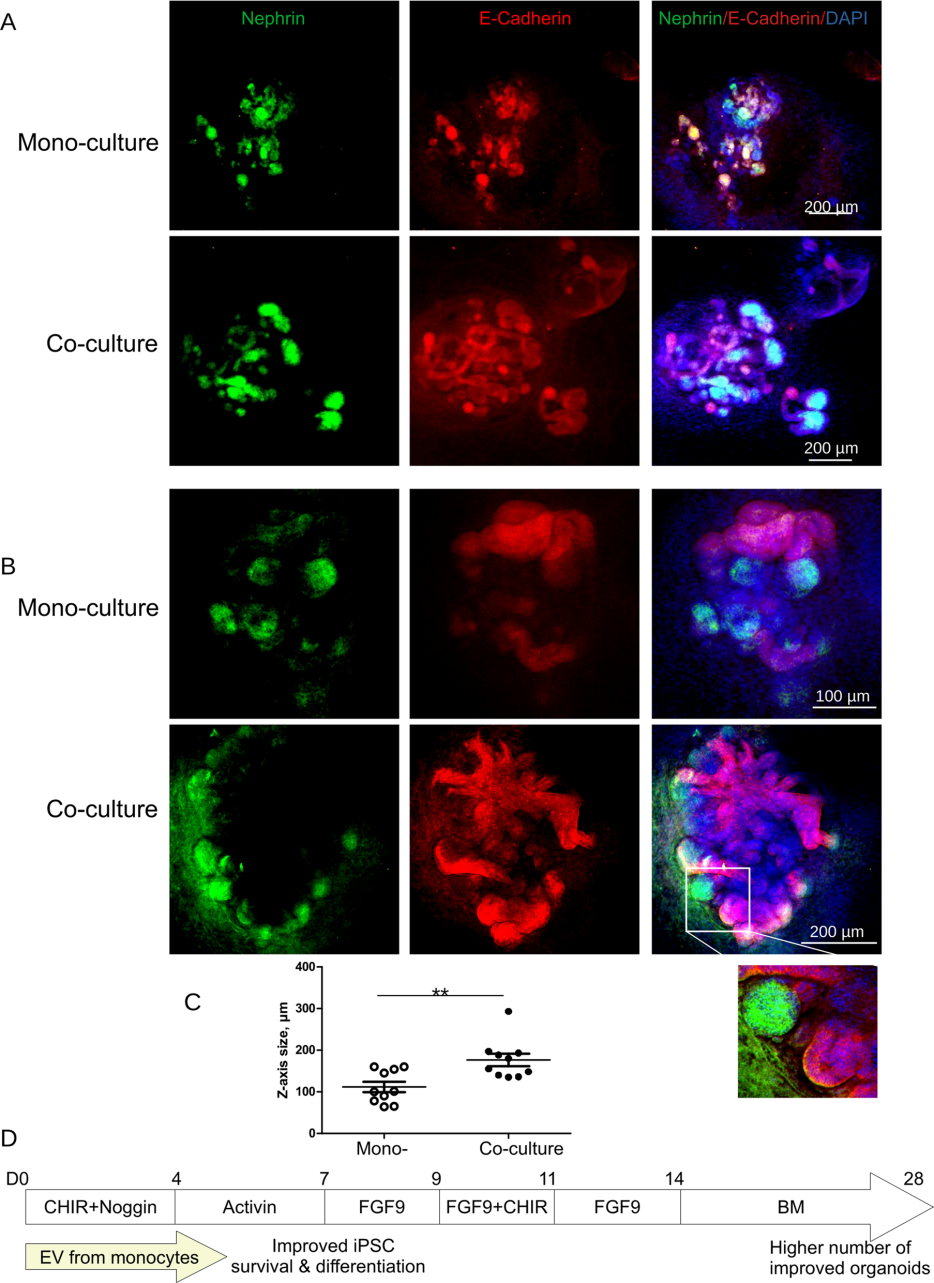
Monocytes promote differentiation of renal organoids. iPSC differentiation to renal organoids has been performed in the transwell co-culture with human monocytes from Day 0 up to the Day 28. **A** and **B** 3D-Confocal microscopy of the renal organoid differentiated in mono culture and in transwell co-culture with human monocytes was performed. **A** For mono- and co-culture 64 and 135 Z-scans, respectively, were imaged with Z-distance of 1 µM. Sum image of Z-scans was obtained using ImageJ. **B** For mono- and co-culture 90 and 140 Z-scans, respectively, were imaged with Z-distance of 1 µM. Right panel shows fragment of one of Z-scans with higher magnification. **C** Size of organoids in z-dimension was analyzed. **D** Schematic diagram of our observations. EVs released by monocytes promote survival and differentiation of iPSC during initial stages of kidney organoids differentiation. This results in higher number and improved development of organoids

We then performed immunohistochemical staining followed by 3D-confocal microscopy, to assess morphological appearance of renal organoids differentiated in co-culture with monocytes (Fig. 6A, B). Two examples of renal organoids differentiated in mono- and co-culture with monocytes and stained using Nephrin and E-cadherin antibodies are shown in Fig. 6A and B. Shown sum of z-scans demonstrate more advanced development of organoids in co-culture with increased size in z-dimension (Fig. 6C). We have performed 3D reconstruction of confocal z-scans using ImageJ. Additional files 4 and 5 videos illustrate improved development of organoids in co-culture. Our data show that EVs released by monocytes promote survival and differentiation of iPSCs during initial phase of the differentiation protocol that results in higher number and better development of iPSCs-renal organoids (Fig. 6).

## Discussion

Developing the iPSCs-derived renal organoids in vitro was a notable advance in generating new approaches with the promise to overcome some disadvantages of animal models [39, 40]. However, kidney organoids are not yet widely accepted as a model for human disease, and applications for drug development appear distant. Further improvement of the differentiation of iPSCs to kidney organoids remains an important and necessary step. Earlier studies focused on the renal developmental signaling pathways, though some aspects of the iPSCs differentiation remain poorly understood. As a result, the role of cell removal (cell death) during organoid development was not appreciated and remains poorly studied. The novel findings of our study could improve that state of affairs by underscoring the role of autophagy and macrophages in kidney organoid development.

During the initial stages of iPSCs differentiation to renal progenitors GSK3β kinase inhibition using CHIR is performed. This treatment results in non-specific activation of Wnt signaling pathway. A similar approach is used during the differentiation of iPSCs to cardiomyocytes. Using two different iPSCs cell lines we observed strong cell death by apoptosis during this step. This was paralleled by increased production of ROS and expression of apoptotic markers cleaved caspase 3 and cleaved PARP-1 (Fig. 3). Our data are consistent with results presented by several groups investigating cardiomyocyte differentiation [24, 26]. Investigators reported that stabilized β-catenin can inhibit both, basal and stress-induced, autophagy [32]. We used the Autophagy detection kit and demonstrated a decreased rate of autophagy rates in the presence of CHIR (Fig. 2C). The importance of autophagy during development and for normal tissue function is undisputed [19]. Activating autophagy by application of rapamycin promoted iPSCs survival and differentiation towards cardiomyocytes [23, 24]⁠. During the differentiation of iPSCs to renal organoids rapamycin improved cell survival by increasing autophagy. However, in contrast to cardiac differentiation, rapamycin could not improve the robust differentiation of renal cells and the formation of kidney organoids.

Our idea was to expand the parameters, as it might be in vivo. We added macrophages into this (complex) equation by co-culturing with human iPSCs-derived macrophages and human blood monocytes. Others observed that tumor-associated macrophages can induce autophagy in the tumor cells thus promoting their survival [21]. However, the mechanism of this effect remains unknown. A recent report pointed out the role of iPSCs-derived microglia in brain organoid development [41]. They showed the role of cholesterol-contained lipid droplets released by microglia-like cells. To investigate whether EVs released by monocytes can promote survival and differentiation of iPSCs, we isolated EVs from monocytes using PEG precipitation. Enriched EVs could indeed promote iPSCs survival and differentiation without monocytes present (Fig. 4). Detailed investigation of monocytes-derived EVs and their cargo remains the objective of further study. The process of autophagy was activated in iPSCs co-cultured with monocytes, similar to the application of rapamycin. Cell survival was also dramatically improved by monocytes and macrophages (Fig. 2). Furthermore, the efficiency of differentiation and the formation of renal organoids has been strongly improved in co-culture with monocytes. One possible explanation for the improved differentiation could be a more efficient removal of damaged mitochondria by the process of autophagy. Such a state of affairs not only could prevent formation of ROS but also may promote the biogenesis of new mitochondria. Mitophagy is a necessary mechanism for mitochondrial biogenesis [42]. During the differentiation of renal organoids, iPSCs switch from glycolysis to oxidative phosphorylation [43] and biogenesis of mitochondria is important for the metabolic reprogramming of the iPSCs. However, it is likely that the role of monocytes is not limited to the activation of autophagy during CHIR stimulation.

There is an opportunity that monocytes in addition to the release of EVs exert also other more basic regulatory functions. We believe it is unlikely that the presence of monocytes can significantly affect the availability of nutrients in the culture because first, the ratio of monocytes to iPSCs was 1:40, respectively; and second, we adjusted the medium volume in co-culture. We have also analyzed the content of L-Lactate in the conditioned media since it reflects the rate of aerobic glycolysis and observed no difference.

Macrophages play an important role during the embryogenesis of the kidney. During organ’s functional development, tissue resident macrophages fulfill anti-inflammatory and repair functions. In vivo, macrophages do not differentiate from the renal progenitors but infiltrate the developing kidney from the yolk sac and fetal liver early in the development [15]. Depletion of macrophages resulted in disturbance of kidney organogenesis and decreased the number of vascular anastomoses in vivo [14]. Our data show that monocytes/macrophages can also promote the differentiation of kidney organoids from iPSCs in vitro. Detailed transcriptomic investigation of kidney organoid differentiation remains the objective of future studies. Nevertheless, our findings provide a novel tool for improving iPSCs differentiation towards kidney organoids.

## Conclusions

Our study showed that the differentiation of iPSCs to kidney organoids can be significantly improved by co-culturing with human classical monocytes. Oxidative stress and apoptosis of iPSCs induced by CHIR are reduced in the presence of monocytes and by the application of rapamycin. Underlying mechanisms imply the activation of autophagy. Furthermore, monocytes but not rapamycin can strongly promote differentiation of iPSCs that results in higher number and better morphological structure of kidney organoids. Our study provides a novel approach for improving the utility of kidney organoid models.

### Supplementary Information

**Additional file 1**. Supplementary figures.

**Additional file 2**. Supplementary Table 1. List of reagents and antibodies used in the study.

**Additional file 3**. Supplementary Table 2. Results of authentication of iPSC cell lines.

**Additional file 4**. Supplementary movie of organoid differentiated in mono-culture.

**Additional file 5**. Supplementary movie of organoid differentiated in co-culture.

## Data Availability

All data generated or analysed during this study are included in this published article and its Supplementary information files.

## Abbreviations

BM: Basal medium
BMP: Bone morphogenetic protein
Carboxy-H_2_DCFDA: 5- (And 6-)Carboxy-2',7'-Dichlorodihydrofluorescein-Diacetate
CCK8: Cell counting kit 8
CHIR: CHIR99021
DPBS: Dulbecco's phosphate-buffered saline
EVs: Extracellular vesicles
FGF: Fibroblast growth factor
FGF9: Fibroblast growth factor 9
Gata3: GATA binding protein 3
GDNF: Glial cell line-derived neurotrophic factor
GSK-3: Glycogen synthase kinase-3
HSP90AB1: Heat shock protein 90 alpha family class B member 1
HSP 70: Heat shock protein 70
IFNγ: Interferon gamma
iPSCs: Induced pluripotent stem cells
IL-4: Interleukin 4
IL-6: Interleukin 6
IL-10: Interleukin 10
LAMP2: Lysosome-associated membrane protein 2
LPS: Lipopolysaccharide
mTOR: Mammalian target of rapamycin
M-CSF: Macrophage colony-stimulating factor also known as colony stimulating factor 1
OSR1: Odd-skipped related 1
PARP1: Poly [ADP-ribose] polymerase 1
p62: Sequestosome-1/ Ubiquitin-binding protein p62
Pax2: Paired box gene 2
PDGFRβ: Platelet derived growth factor receptor beta
PEG: Polyethylenglycol
PVDF: Polyvinylidene difluoride
ROS: Reactive oxygen species
SDS: Sodium dodecylsulfate
TBX6: T-Box transcription factor 6
TNFɑ: Tumor necrosis factor alpha
Wnt: Wingless/Integrated
