# Supplementary material for: Monocytes prevent apoptosis of iPSCs and promote differentiation of kidney organoids
Source: Stem Cell Res Ther. 2024 May 3;15:132. doi: 10.1186/s13287-024-03739-8 (PMC11069262; doi:10.1186/s13287-024-03739-8)
Supplement: Supplementary file 1 — Additional file 1. Supplementary figures. [file 13287_2024_3739_MOESM1_ESM.pdf]

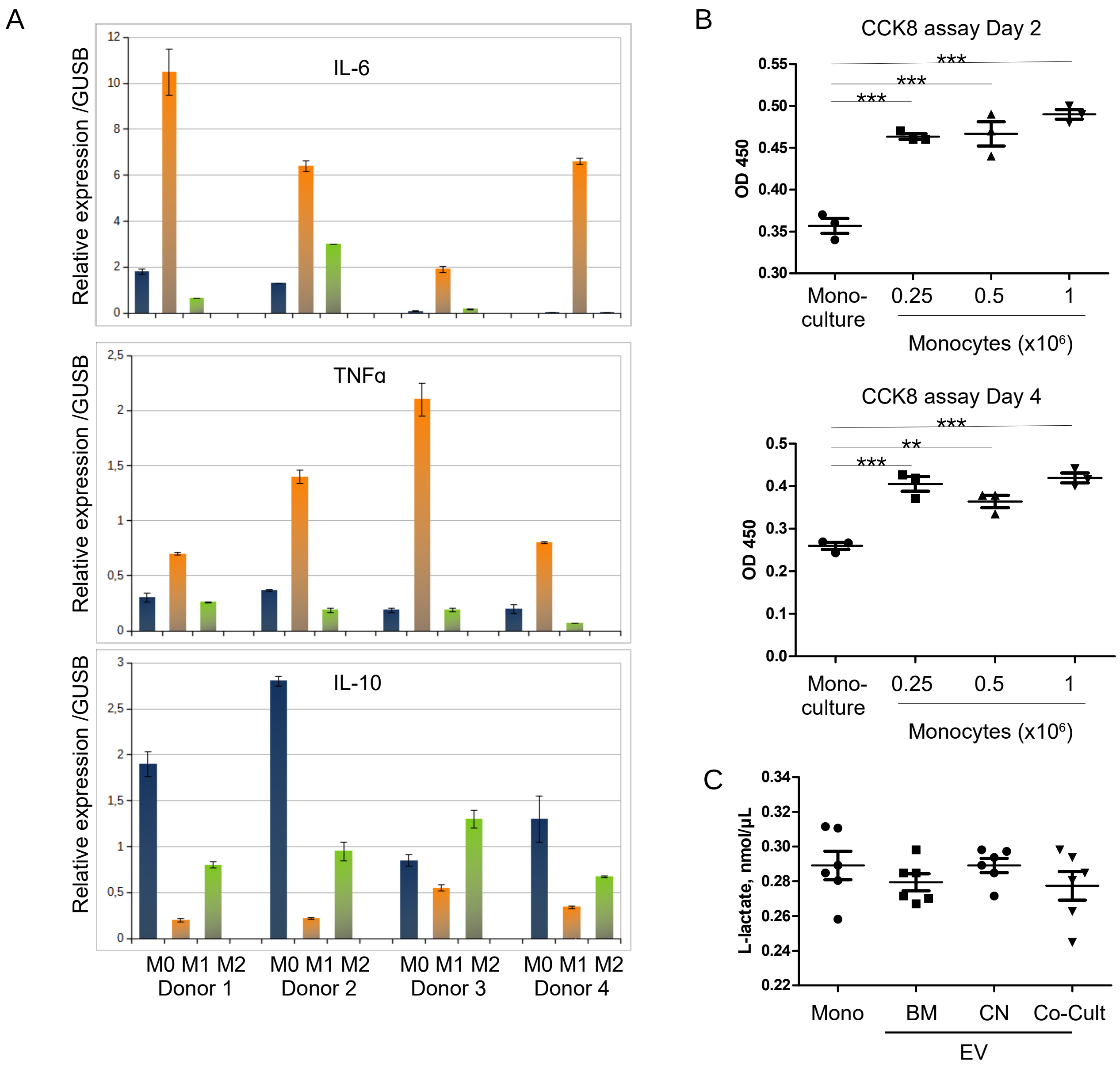

Supplementary figure 1. Characterization of monocytes functionality and optimization of monocytes/iPSC ratio. A. Human monocytes isolated from 4 different donors were differentiated to macrophages in the presence of M-CSF and then polarized to M1- and M2-like macrophages. For M1-like polarization, IFNγ and LPS stimulation was used, for M2-like polarization IL-4 was used. Expression of IL-6, IL-10 and TNFα was used to characterize polarization of macrophages. B. iPSC differentiation to kidney organoids was induced in mono-culture and in transwell co-culture with different amount of isolated monocytes. CCK-8 analysis was performed after 2 and 4 days. C. Concentrations of L-Lactate were measured in conditioned medium of iPSC treated with EV from monocytes analyzed at day 4.

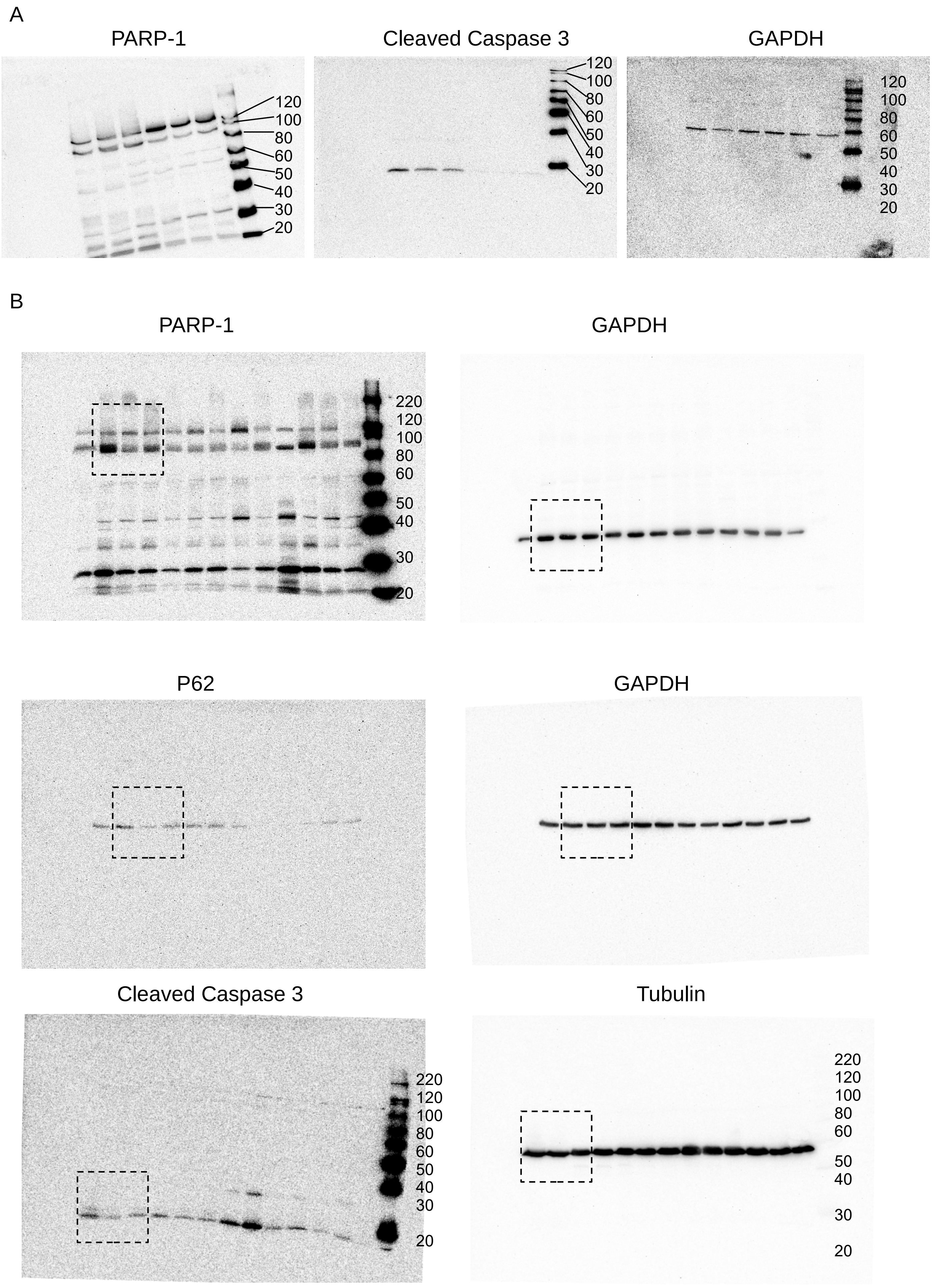

Supplementary figure 2. Uncropped gels for Figure 1 C (A) and Figure 3 B-D (B).

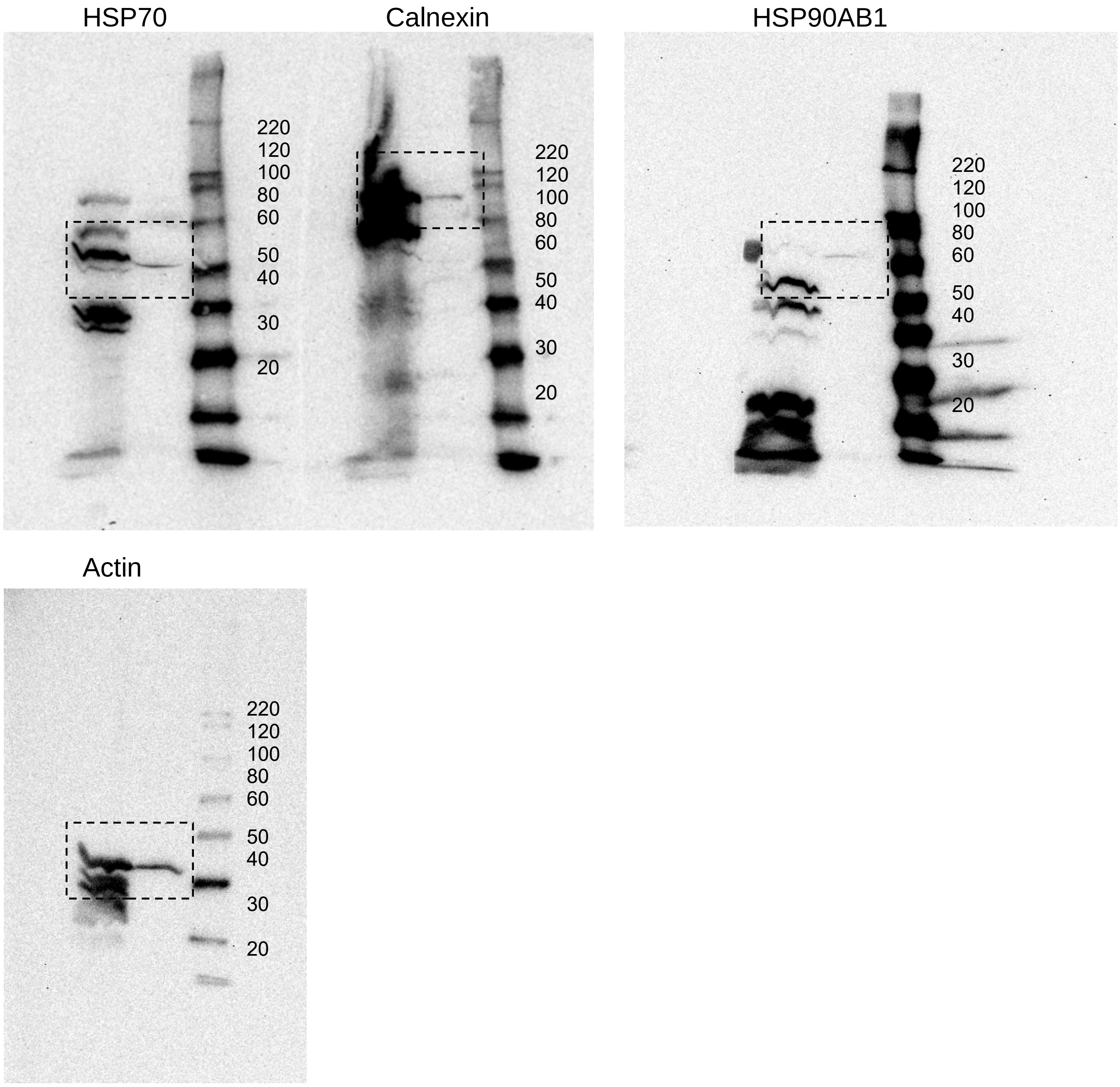

Supplementary figure 3. Uncropped gels for Figure 4B.
