# Supplementary material for: Monocytes prevent apoptosis of iPSCs and promote differentiation of kidney organoids
Source: Stem Cell Res Ther. 2024 May 3;15:132. doi: 10.1186/s13287-024-03739-8 (PMC11069262; doi:10.1186/s13287-024-03739-8)
Supplement: Supplementary file 2 — Additional file 2. Supplementary Table 1. List of reagents and antibodies used in the study. [file 13287_2024_3739_MOESM2_ESM.pdf]

SCRT-D-23-01518

Supplementary Table 1. List of reagents and antibodies used in the study.

Reagent	Vendor	Catalog #
**Cell culture**		
Human Episomal iPSC Line	Gibco by ThermoFisher	A18945
ED- iPSC cell line	CEA, Institute Joliott, Paris, France	
StemFlex	Gibco by ThermoFisher	A3349401
DPBS	Gibco by ThermoFisher	14190144
StemPro Accutase	Gibco by ThermoFisher	A1110501
DMEM/F12	Gibco by ThermoFisher	11320033
Cell culture plates	CELLSTAR	657160
Geltrex	Gibco by ThermoFisher	A1413302
Y-27632 2HCl ROCK inhibitor	Selleckchem	S1049
Laduviglusib (CHIR-99021) HCl	Selleckchem	S2924
Rapamycin	Selleckchem	S1039
Advanced RPMI 1640 medium	PAN-Biotech	P04-16500
Fetal Bovine Serum	PAN-Biotech	P30-3306
Cell culture flasks	SARSTEDT	83.3911.002
Classical Monocyte Isolation Kit	Miltenyi Biotec	130-117-337
Lymphocytes-Separation Medium 1077	PromoCell	C-44010
Leucosep tubes	Greiner CELLSTAR by Merck	227290
Human Activin A Recombinant Protein	PeproTech by ThermoFisher	120-14E-10UG
L-Glutamine	PAN-Biotech	P04-80100
KnockOut Serum Replacement	Gibco by ThermoFisher	10828028
Recombinant Human Noggin	PeproTech by ThermoFisher	120-10C

Human Activin A Recombinant Protein	PeproTech by ThermoFisher	120-14E
Recombinant Human FGF-9 Protein	R&D Systems, Inc., a Bio-Techne Brand	273-F9-025
Human recombinant M-SCF	PeproTech by ThermoFisher	300-25
Human recombinant IL-4	PeproTech by ThermoFisher	200-04
LPS	Sigma	L2887
Human recombinant IFNγ	PeproTech by ThermoFisher	300-02
Transparent Thincert cell culture inserts	Greiner Bio-One	657641 or 662641
Nunc 96-Well Optical Coverglass Bottom plate	Revvity	6005182
ethanol	J.T.Baker by Avantor	8025
**RT-PCR**		
The Qiagen RNeasy Mini Kit	Qiagen	74106
β-mercaptoethanol	Sigma-Aldrich by Merck	M3148
Pax2	ThermoFisher Scientific	HS01057416_m1
Gata 3	ThermoFisher Scientific	HS00231122_m1
Nephrin	ThermoFisher Scientific	HS00190446_m1
Cdh1	ThermoFisher Scientific	OS01023895_m1
PDGFRβ	ThermoFisher Scientific	HA01019589_m1
TBX6	ThermoFisher Scientific	Hs00365539_m1
OSR1	ThermoFisher Scientific	Hs00377071_m1
IL-6	ThermoFisher Scientific	Hs00985639_m1
IL-10	ThermoFisher Scientific	Hs00961622_m1
TNFa	ThermoFisher Scientific	Hs00174128_m1
GUSB	ThermoFisher Scientific	HS00939627_m1
LightCycler® 480 RNA Master Hydrolysis Probes	Roche	04991885001
**Assays and kits**		

Autophagy CYTO-ID Autophagy detection kit	Enzo	ENZ-51031-0050
Cell Counting Kit-8	Dojindo	CK04-13
BioTracker 488 Green Mitochondria Dye	Millipore by ThermoFisher	SCT136
5- (und 6-)Carboxy-2',7'-Dichlordihydrofluorescein-Diacetat (Carboxy-H2DCFDA)	ThermoFisher	C400
CellRox green reagent	ThermoFisher	C10444
MitoSOx green reagent	ThermoFisher	M36006
**Reagents**		
Albumin, Human, Fraktion V, pH 7,0	SERVA	11930.04
Paraformaldehyd	SERVA	31628
Goat serum	Sigma-Aldrich by Merck	G9023
Donkey serum	Sigma-Aldrich by Merck	D9663
Sodium orthovanadate	Sigma-Aldrich by Merck	S6508
Tween 20	Carl Roth	9127.1
Triton™ X-100	Sigma-Aldrich by Merck	9002-93-1
Glycine	Carl Roth	3187.4
SDS pellets	Carl Roth	8029.4
Methanol	J.T.Baker by Avantor	67-56-1
PVDF Western Blotting Membranes	Sigma-Aldrich by Merck	03010040001
Phenylmethylsulfonylfluorid	Carl Roth	6367.1
TRIS Hydrochlorid	Carl Roth	9090.2
Natriumchlorid	Carl Roth	0601.2
Crystal Violet	Sigma-Aldrich by Merck	548-62-9
Ethylenediaminetetraacetic acid disodium salt dihydrate	Sigma-Aldrich by Merck	6381-92-6
Sodium deoxycholate	Sigma-Aldrich by Merck	302-95-4
Leupeptin trifluoroacetate salt	Sigma-Aldrich by Merck	147385-61-3

Aprotinin from bovine lung	Sigma-Aldrich by Merck	9087-70-1
**Antibody**		
Cleaved Caspase-3 (Asp175) (5A1E) Rabbit mAb	Cell Signaling Technology	9664
PARP Antibody	Cell Signaling Technology	9542
SQSTM1/p62 Antibody (D-3)	Santa Cruz Biotechnology	sc-28359
GAPDH Antibody (6C5)	Santa Cruz Biotechnology	sc-32233
HSP90AB1		
HSP70		
Calnexin		
Actin		
Nephrin	R&D Systems	AB_2154851
E-Cadherin	Abcam	Ab11512
Donkey anti-Sheep IgG (H+L) Cross-Adsorbed Secondary Antibody, Alexa Fluor™ 488	ThermoFisher	A-11015
Goat anti-Mouse IgG (H+L) Highly Cross-Adsorbed Secondary Antibody, Alexa Fluor™ Plus 647	ThermoFisher	A32728
Anti Mouse IgG HRP-linked	Cell Signaling	7076S
Anti Rabbit IgG HRP-linked	Cell Signaling	7074S
Anti Goat IgG HRP-coated	R&D Systems	HAF109
DAPI (4',6-Diamidino-2-Phenylindole, Dihydrochloride)	ThermoFisher	D1306
