# Supplementary material for: Monocytes prevent apoptosis of iPSCs and promote differentiation of kidney organoids
Source: Stem Cell Res Ther. 2024 May 3;15:132. doi: 10.1186/s13287-024-03739-8 (PMC11069262; doi:10.1186/s13287-024-03739-8)
Supplement: Supplementary file 3 — Additional file 3. Supplementary Table 2. Results of authentication of iPSC cell lines. [file 13287_2024_3739_MOESM3_ESM.pdf]

Eurofins Genomics Europe Food/Environment/White Biotech Products & Services GmbH, Anzinger Str. 7 a, D-85560 Ebersberg

eurofins | Genomics

Analytical Report:
Cell Line Authentication Test
Order ID: 11108795952

Method:
DNA isolation was carried out from cell pellet (cell layer).
Genetic characteristics were determined by PCR-single-locus-technology.
16 independent PCR-systems D8S1179, D21S11, D7S820, CSF1PO, D3S1358, TH01, D13S317, D16S539, D2S1338, AMEL, D5S818, FGA, D19S433, vWA, TPOX and D18S51 were investigated.
(ASN-0002 core markers are colored grey, Thermo Fisher, AmpFlSTR® Identifiler® Plus PCR Amplification Kit)
In parallel, positive and negative controls were carried out yielding correct results.
Method details are given in **SOP_APG_Zelllinienauthentizität_2.0**

Result:

Client Sample Name	ED-IPSC 1	EPI-IPSC1
**Sample Code**	CL00016893	CL00016894
D8S1179	13,13	9,15
D21S11	30,33.2	29,29
D7S820	9,11	10,12
CSF1PO	9,12	11,11
D3S1358	16,17	15,18
TH01	6,9	9,9.3
D13S317	10,11	9,11
D16S539	12,13	11,13
D2S1338	20,21	17,24
D19S433	13,13	12,14
vWA	17,18	17,18
TPOX	8,9	8,8
D18S51	12,16	15,15
AMEL	X,X	X,X
D5S818	10,11	11,12
FGA	22,23	22,25

The laboratory is accredited acc. to **DIN EN ISO/IEC 17025:2018**. All analyses have been carried out with greatest care and on the basis of state of the art scientific knowledge. The results refer solely to the analysed samples, as received. The duplication and publication also in parts requires a written authorization by this laboratory. Our General Terms and Conditions apply exclusively and are available under eurofinsgenomics.com

ilac-MRA DAkkS Deutsche Akkreditierungsstelle D-PL-13372-01-00

This report was created automatically and is therefore valid without a signature.

Eurofins Genomics Europe Food/Environment/White Biotech Products & Services GmbH
Anzinger Straße 7 a
85560 Ebersberg
Germany

Tel.: +49 7531 816068
Fax: +49 8092 21084

Email: support-eu@genomics.eurofinseu.com
Web: eurofinsgenomics.com

Managing Directors: Dr. Mashal Alawi & Dr. Sonja Wiedemann

Register Court Munich HRB 207710
VAT ID: DE815473648

HypoVereinsbank

IBAN: DE23 2073 0017 7000 0006 50
SWIFT: HYVEDEMME17
